# Relationship between blood group (ABO) and risk of COVID-19 infection in a patient cohort in Tehran, Iran

**DOI:** 10.1099/acmi.0.000544.v5

**Published:** 2024-06-26

**Authors:** Asal Fathollahi, Haniyeh Bashizadeh Fakhar, Babak Shaghaghi

**Affiliations:** 1 Department of Medical Science, Chalus Branch, Islamic Azad University, Chalous, Iran; 2 Department of Laboratory Science, Chalous Branch, Islamic Azad University, Chalous, Iran

**Keywords:** ABO blood type, COVID-19, infection

## Abstract

**Background and purpose.** Coronavirus (COVID-19) is a contagious disease causing severe acute respiratory syndrome which had a catastrophic effect on the world population and resulted in more than 2.9 million deaths worldwide. Epidemiological investigations have recently announced blood type has an association with the incidence of COVID-19 infection. Consequently, research in this regard can be effective in determining a person’s susceptibility to a viral infection. Therefore, we investigated the relationship between blood types and the risk of COVID-19 in patients admitted to Khorshid laboratory, Tehran, Iran.

**Materials and methods.** From January to March 2020, 50 nasal and throat swapb samples of patients’ secretions were obtained from patients who were admitted to Khorshid laboratory. They were confirmed to have COVID-19 virus RNA and real-time polymerase chain reaction (PCR)-ABI, and their blood type was determined simultaneously. After collecting data to determine the relationship between COVID-19 infection and blood type, a confidence interval of 90 % was considered using SPSS 16.

**Results.** The mean age of the patients was measured at 38.4±6.3 years. According to PCR results, 100 % of the subjects with COVID-19 showed blood type A. In addition, the ratio of blood type A to the percentage of reference type O was higher (*P*=0.009).

**Conclusion.** There was a significant relationship between ABO blood type and susceptibility to COVID-19. As the current study suggests, those with blood type A are at a higher COVID-19 infection risk than those with blood type O.

## Data Summary

We have not generated no data during this research nor is any required for the work to be reproduced.

## Introduction

In late December 2019, an unidentified case of pneumonia was reported in Wuhan, China. World Health Organization (WHO) and the International Committee for the Classification of Viruses called it COVID-19 and SARS-COV2, respectively. Early genomic analysis of the virus, confirmed its homology to the coronavirus bat SARS (SARS-COV.RATG13) [1]. Coronavirus ais a highly contagious disease causing severe acute respiratory syndrome which has had a devastating impact on the global population, resulting in more than 2.9 million fatalities worldwide. The United States had the highest rate of infection and deaths due to COVID-19 [2]. Coronavirus is a single-stranded RNA virus (diameter: 80–120 nm) and is divided into four groups of alpha, beta, delta, and gamma [3]. Due to the coronavirus adaptation in the bat body with a higher temperature compared to the human body, this virus is highly resistant against temperature in comparison with SARS.COV [4,5]. High genetic diversity as well as repeated recombination of coronavirus have enhanced its interspecific transmission [6]. Respiratory droplets and direct contact are the most frequent methods of the virus transmission. Its latency period is an average of 3 days and the mean duration from the first symptom onset to death is 14 days) [7]. After entering the body, the CovidOVID-19 virus targets the lung tissue, attaching by branches on its spherical covering to a receptor called ACE2 on some cells [8,9]. Fever, fatigue, vomiting, cough, and diarrhoea are the most prevalent clinical symptoms. Besides the lungs, the virus affects other tissues, such as the liver, heart, eyes, kidneys, and nervous system [10]. Unmarked carriers are important players in the transmission of the disease between individuals, and the best method for diagnosing this group is the real-time polymerase chain reaction (RT.PCR) [11]. Based on the Chinese Centre for Disease Control and Prevention (CCDC) reports on cardiovascular disease, the groups most sensitive' group to the virus are people with diabetes and high blood pressure, chronic respiratory diseases, and cancer [12,14]. According to reports, the viral death rate is 3.4 % [15]. Previous studies on the relationship between ABO blood types and host vulnerability against infectious diseases, such as SARS-COV [16], malignant tumours [17], Norwalk virus [18], and Helicobacter pylori [19] has been identified as hepatitis B virus [20]. Epidemiological investigations have recently declared that blood type has a strong association with the incidence of COVID-19 infection [21]. Over the years, research on the relationship between blood types and viral infections has been considered. Therefore, research in this regard can be effective in determining a person’s susceptibility to viral infections. Consequently, we investigated the relationship between blood types and the risk of COVID-19 infection in cases admitted to Baqiyatallah Hospital, Tehran, Iran.

## Methods

50Fifty patients hospitalized in Shohada Tajrish Hospital from December to March 2020, whose tests were performed in Khurshid Laboratory, Tehran, Iran, participated in the study. The Ethics Committee of Azad University of Medical Sciences approved the method of the current study. Moreover, all patients were consulted by an infectious disease specialist before sampling. Firstly, a skilled nurse from each 50 patients took one tube citrated blood sample (equal to 2 ml) and one sterile swapb from throat and nose secretions. The samples were quickly transferred to the laboratory under sterile conditions (for whole blood we used citrate’s tubes and for throat’s swapbs we used viral transport media (VTM). Then, after being molecularly transmitted and homogenized, they were stored at −20 °C. Then, blood samples were transferred into tubes containing citrate and referred to a haematology laboratory. Subsequently, based on the standard method of antigen agglutination test (useding American Association of Blood Banks [AABB] Technical Manual), the antibody was determined. Isolation of COVID-19 RNA from secretions was performed using the RNA Roche extraction kit and finally, RT-PCR by the Italian-made Sacacce kit. Then, the results were displayed at the same time by the Real-time ABI device.

## Statistical analysis

The statistical analysis in the provided text includes the utilization of various methods. Firstly, we opted for a 90 % confidence interval (CI) to estimate COVID-19 prevalence using Real-Time PCR and blood group analysis in a sample of 50 individuals. This choice balances precision with the risk of error, offering a reasonably high degree of confidence in the estimated prevalence. While narrower than higher confidence levels, like 95 % or 99 %, a 90 % CI still ensures statistical robustness. Accepting a 10 % risk of the estimate falling outside the interval allows for more precise estimates while maintaining flexibility and practicality given our sample size (50 cases) and data constraints. Mean and Standard Deviation (SD) were reported to convey the average and data spread, while One-Way Analysis of Variance (ANOVA) was used for inter-group comparison. Additionally, all data analysis was conducted using SPSS 16 software, with a significance level considered for tests with a p-value less than 0.05. These methods collectively ensure robust statistical analysis while addressing the specific constraints and objectives of the study.

## Results

The subjects’ mean age was measured at 38.4±6.3 years. The patients' age ranged from 22 to 53 years. Based on the PCR test, of 50 swab samples from the nasopharyngeal secretions of COVID-19 patients, 66 % were tested positive and 34 % as negative. Based on the obtained information, according to specific diseases and their symptoms, 16 % of subjects had a fever, (48 %), cough, (28 %), gastrointestinal symptoms, (28 %), sluggishness, and lethargy (54 %). The distribution of the subjects’ O.A.AB.B blood types were (24, 28, 20, 28 %), respectively. Around 26 % of the samples were Rh-negative and 74 % were Rh-positive and the comparison of Rh blood types was not significantly related (*P*=0.086). According to the PCR test results, all the patients infected with COVID-19 had blood type A, and blood type AB, B, and O reported to be 60 %, 64 %, and 33 %, respectively. That ratio of blood type A to the percentage of reference group O was higher (*P*=0.009), which was statistically significant (Table 1).

**Table 1.** Comparison of PCR percentage by blood groups

		PCR									
		Negative		Positive		Total			95 % CI OR		
		N	Row N %	N	Row N %	N	Row N %	P	Odds ratio	LowerCL	Upper CL
Blood group	A	0	0	14	100	14	100	0.009	54.8	2.62	1 147.43
	AB	4	40	6	60	10	100	0.217	3	0.152	17.15
	B	5	35.7	9	64.3	14	100	0.122	3.6	0.709	18.25
	O	8	66.7	4	33.3	12	100	Reference group			
	Total	17	34	33	66	50	100				
RH	Negative	7	53.8	6	46.2	13	100	Reference group			
	Positive	10	27	27	73	37	100	0.086	3.15	0.85	11.67
	Total	17	34	33	66	50	100				

The following table illustrates the PCR test results, indicating people with COVID-19 showed more severe symptoms of fever, cough, sluggishness, and lethargy (*P*<0.001), which is statistically significant (Table 2).

**Table 2.** Comparison of PCR percentages by symptoms and specific diseases

		PCR				
		Negative		Positive		
		Count	Row N %	Count	Row N %	P=
Fever	No	16	80	4	20	<0.001
	Yes	1	3.3	29	96.7	
	Total	17	34	33	66	
Cough	No	17	65.4	9	34.6	<0.001
	Yes	0	0	24	100	
	Total	17	34	33	66	
Gastrointestinal symptoms	No	14	38.9	22	61.1	0.242
	Yes	3	21.4	11	78.6	
	Total	17	34	33	66	
Sluggishness and lethargy	No	16	69.6	7	30.4	<0.001
	Yes	1	3.7	26	96.3	
	Total	17	34	33	66	
Specific disease	No	17	40.5	25	59.5	0.027
	Yes	0	0	8	100	
	Total	17	34	33	66	
Age	Under 40 years	13	44.8	16	55.2	0.058
	Upper 40 years	4	19	17	81	
	Total	17	34	33	66	

## Discussion

With the advent of coronavirus for the first time in Wuhan, China, and then, in a short time, its spread to all countries around the world, the WHO (World Health Organization) called it COVID-19, followed by a pandemic and the declaration of a state of emergency. COVID-19 is a newly found virus, and despite the various studies conducted in its regard so far, many of its dimensions remain unknown [22]. Infection with COVID-19 has been associated with a variety of variables, including male gender, old age, diabetes, asthma, and other medical conditions [23,24]. Epidemiological investigations have declared that blood type is highly proportional to COVID-19 infection [21]. The ABO histo-blood system is constituted of three antigens (ABH) and the addition of carbohydrate units in the precursor column of oligosaccharides leads to the formation of four AB.B.A.O phenotypes [25,27]. The Rh blood type depends on the presence or absence of protein D epitope, which is classified into two phenotypes of D positive and D [26,27]. ABO blood group antigens are found in superficial lymphocytes, red blood cells, as well as many tissue organs, mucosal surfaces, and exocrine secretions [25,28, 29]. These antigens affect the immune system and, using natural host antibodies and complementary systems, can affect the spread of pathogens [25,28, 29]. Various studies have been performed on the relationship between the biological functions of different viruses with blood type and their association with rotavirus, neuro viruses, dengue virus, Norwalk virus, and hepatitis B [30,31]. Researchers discovered that O-glycosylation, or the binding of carbohydrates to the oxygen group in proteins, had a major role in the dissemination of the SARS infectious pandemic. Researchers have found that a similar scenario may occur, and that glycoprotein O is a critical component of the infection [32,33].

According to epidemiological studies, blood type has a strong association with the incidence of COVID-19 infection [21]. A meta-analysis study conducted in April 2020 in Sari, Iran, on 318 studies carried out to investigate the relationship between ABO blood type and the incidence of COVID-19, revealed that those with blood type A were at higher risk of infection, whereas cases with blood type O were at lower risk of infection [34]. Another research in China, which reviewed 715 studies revealed that blood types B and A are possibly a risk factor for COVID-19 contraction, while blood type O was at lower infection risk [35]. In another research carried out in Afghanistan in 2021, a total of 301 people with positive RT.PCR for COVID-19 were compared to 1 039 people in the control group, their result showed that the incidence of COVID-19 infection in people with blood type A was higher [36]. Belaone and colleagues examined COVID-19 in a population of 1 094 people in Morocco in December 2020, after RT.PCR positive results were observed in 242 patients, blood type A was the highest blood type among COVID-19 patients, while blood type O was the lowest [37]. Additionally, since March 2020, several studies concerning the relationship between COVID-19 and ABO blood type were conducted, including (Zhao *et al*. [38], Zitz *et al*. [39], Zang *et al*. [40], Li *et al*. [41], and Wu *et al*. [42]). All the aforementioned studies confirm that those with blood type A are more at risk of COVID-19 infection than cases with blood type O. In the current study on 50 samples, 33 of which were COVID-19 positive as evident by their positive PCR test. Their blood type results showed that 100 % of patients with blood type A had COVID-19, whereas patients with blood type O had the lowest COVID-19 incidence rate, which was consistent with the findings of previous studies. However, there was no significant relationship between Rh blood type and COVID-19 contraction. In a study carried out in March 2020 in Tehran, Iran, a review of 397 RT. PCR-positive individuals reported a lower COVID-19 infection risk in those with blood type O, while Rh blood types were not significantly associated with the rate of COVID-19 infection [43]. In another study performed on 242 patients, who werehad tested positive forby RT.PCR, it was found that the ratio of blood type A in patients with COVID-19 was markedly higher compared to the control group. Moreover, there was no significant correlation between Rh blood types [44]. Symptoms of coronavirus include respiratory disorders, sore throat, dry cough, runny nose, dizziness, and body aches that are accompanied by headache and fever. The aforementioned symptoms in the elderly, children, and people with disabilities can become more severe and cause bronchitis [45,46]. In the present study according to positive PCR result in cases in terms of fever (20 %) vs. (96.7 %) (P:0.001) Ccough (100 %) vs. (34.6) (*P*<0.001) sluggishness and lethargy (*P*<0.001). It was statistically significant and in the mentioned cases, the severity of symptoms was reported. In December 2020, a study of 1 816 patients with more severe symptoms and shortness of breath was chest pain and cough [47]. In another study in 2020 in China, which was performed on 655 COVID-19 cases, symptoms such as dry cough, fatigue, muscle pain, and fever significantly impact (*P*<0.001) [48]. Another study in 2020 collected data from 14 eligible research, including 1 424 patients, who were assessed and showed symptoms. The cough and fever were reported to be significantly higher than the rest of the symptoms [49]. Another study was performed on 14 families and their children in China, showing that the symptoms of cough, fever, and pain were the most severe among children [50], which was consistent with the results obtained from the parents. Certainly, the current study has limitations, and more research in this field is needed to acquire additional information.

## Conclusion

As the results of previous studies and the present research revealed, there is a marked association between ABO blood type and there is a Rrisk of infection with COVID-19. Therefore, the current study showed those with blood type A are more prone to contracting COVID-19 compared to people with blood type O. The distribution of blood types in the population can lead to a better understanding of the epidemic conditions. Health policies are useful to reduce the spread of this virus.

It is clear that more research is needed. Further studies should also be performed on a larger community of patients with coronavirus infection in this geographical area. Further studies are required to determine the relationship between ABO blood types and susceptibility against COVID-19.

## Abbreviations

ABO: blood gruop
COVID-19: coronaviruse
PCR: polymerase chain reaction
RT.PCR: reverse transcription polymerase chain reaction
WHO: World Health Organization
